# Exploring the mechanism of action of *Vanda tessellata* extract for the treatment of osteoarthritis through network pharmacology, molecular modelling and experimental assays

**DOI:** 10.1016/j.heliyon.2024.e35971

**Published:** 2024-08-08

**Authors:** Sucheesmita Padhee, Debajani Mohanty, Ambika Sahoo, Sudipta Jena, Pratap Chandra Panda, Asit Ray, Sanghamitra Nayak

**Affiliations:** Centre for Biotechnology, Siksha O Anusandhan (Deemed to be University), Kalinganagar, Ghatikia, Bhubaneswar, India

**Keywords:** *Vanda tessellata* extract, Molecular docking, MD simulation, Network pharmacology, Osteoarthritis

## Abstract

The present study employed a comprehensive approach of network pharmacology, molecular dynamic simulation and *in-vitro* assays to investigate the underlying mechanism of the anti-osteoarthritic potential of *Vanda tessellata* extract (VTE). Thirteen active compounds of VTE were retrieved from the literature and the IMPPAT database. All of these passed the drug likeness and oral bioavailability parameters. A total of 535 VTE targets and 2577 osteoarthritis related targets were obtained. The compound-target-disease network analysis revealed vanillin, daucosterol, gigantol and syringaldehyde as the core key components. Protein-protein interaction analysis revealed BCL2, FGF2, ICAM 1, MAPK1, MMP1, MMP2, MMP9, COX2, STAT3 and ESR1 as the hub genes. Kyoto Encyclopedia of Genes and Genomes (KEGG) enrichment analysis revealed AGE-RAGE signalling pathway, HIF-1 signalling pathway and ESR signalling pathway as the major signalling pathway of VTE involved in treating osteoarthritis. Molecular docking analysis showed daucosterol and gigantol to have good binding affinity with BCL2, ESR1 and MMP9, and the results were further confirmed through molecular dynamics simulation analysis. The mechanism predicted by network pharmacology was validated *in vitro* on IL-1β-induced SW982 synovial cells. VTE did not show any cytotoxicity and inhibited the migration of SW982 cells. VTE inhibited the expression level of IL-6, IL-8, TNF-α, PGE-2, MMP-2 and MMP-9 in a dose-dependent manner. VTE inhibited nuclear translocation of NF- κβ and suppressed phosphorylation of p38, extracellular signal-regulated kinase (ERK), and c-Jun NH2-terminal kinase (JNK) of the mitogen-activated protein kinase (MAPK) signalling pathway. The results showed that VTE exerted an anti-osteoarthritic effect by a multi-target, multi-component and multi-signalling pathway approach.

## Graphical abstract

**Figure:**
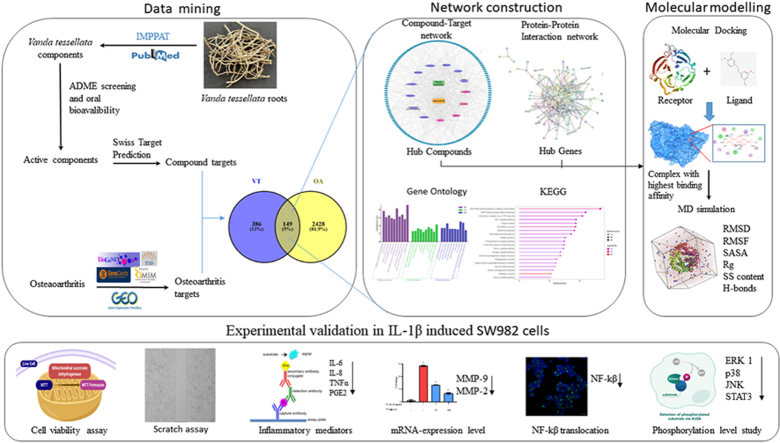

## 1 Introduction

Osteoarthritis (OA) is one of the most prevalent degenerative age-related joint diseases, affecting nearly 300 million individuals worldwide [1]. It affects almost every joint of the body, including the knees, feet, hands, and hips and is characterised by joint discomfort, swelling, and stiffness, which can hamper a person's ability to carry out their routine work [2,3]. The clinical characteristics of OA include the breakdown of joint cartilage, thickening of subchondral bone, osteophytes development, inflammation of the synovial lining, internal joint inflammation, and changes in the structure of the joint capsule, ligaments, and surrounding muscles [[4], [5], [6]]. OA risk factors include lifestyle choices, dietary habits, aging, genetic predisposition, obesity, and mechanical injuries [7,8].

Currently, there is no cure for OA, and the drugs available in the market primarily focuses on symptoms management rather than addressing the underlying cause. Drug therapies like non-steroidal anti-inflammatory drugs (NSAID) or selective COX-2 inhibitors are frequently employed to alleviate joint pain. However, their prolonged use often leads to gastrointestinal complications [9,10]. Intra-articular corticosteroids offer a strong anti-inflammatory effect, which helps to reduce joint pain and swelling but exhibits extensive side effects [11]. TNF-α, IL-1 and IL-6 antagonists show promising therapeutic effects, yet they make the body more prone to inflammation and induces tumour-related diseases [12,13]. Hence, there is an urgent need to develop a drug with significant curative properties devoid of any side effects.

For centuries, plants have been utilised for their healing properties, offering a vast range of beneficial compounds that have traditionally been used without causing any side effects [14]. Plant-derived products have been reported to alleviate osteoarthritis symptoms [15]. The medicinal values of many orchid species have been recognized since ancient times [16]. Many plants are also reported for their efficiency in treating arthritis in ayurvedic system of medicine [17]. Bioactive compounds derived from orchids possess high medicinal potential and can act as a source of drugs [18].

*Vanda tessellata* (Roxb.) Hook. ex G.Don (Syn.*Vanda rouxburghii*) is an epiphytic subshrub of the family Orchidaceae and is distributed across different parts of India, Nepal, Bangladesh, Burma, Vietnam, China and Sri Lanka [19]. In the Unani system, the plant juice is used for treating toothaches, bronchitis, boils and as a tonic for the brain and liver [20]. Several pharmacological properties of *Vanda tessellata* such as anti-inflammatory, anti-convulsants, anti-diarrhoea, antioxidant, anti-microbial, wound healing, neuroprotective and hepatoprotective activity, have also been previously reported [[21], [22], [23], [24], [25], [26], [27], [28]]. Different parts of the plant are used to treat pain, inflammation, arthritis, sciatica, liver disease, bronchitis, hiccough, and fever [29]. The root of *Vanda tessellata* is reported to exhibit activity against bacterial infection, tuberculosis, abdominal diseases, tremors, and otitis [30]. Root & leaf extracts of *Vanda tessellata* possess significant analgesic and anti-inflammatory activity [28]. Phytochemical screening studies have indicated the presence of different chemical classes such as polyphenols, alkaloids, terpenoids, flavonoids, tannins, fatty acids, saponins, glycosides and sitosterol in the plant [31].

The conventional drug discovery approach has some limitations due to the complex nature of diseases that involve interaction among various molecules and pathways. Hence, targeting a single molecule might not be enough to treat a disease effectively. Network pharmacology is an emerging area of study that uses complex interaction networks to explore complex biological system and their interaction with the drug [32,33]. Although a previous study on an arthritic rat model has suggested the potency of *Vanda tessellata* against arthritis [34], the underlying molecular mechanisms is still not clear. Additionally, there is a lack of a comprehensive understanding of the pharmacodynamic properties of the active constituents of *Vanda tessellata* and the primary molecular targets responsible for its anti-osteoarthritic effects. Therefore, the present study aimed to investigate the potentiality of *Vanda tessellata* extract (VTE) for treating OA through a comprehensive approach using network pharmacology, molecular docking, molecular dynamic simulation and *in-vitro* assays.

## 2 Materials and methods

### 2.1 Network pharmacology

#### 2.1.1 Identification and screening of drug-like candidates of Vanda tessellata

The active chemical constituent of VTE was retrieved the literature [35] and the Indian Medicinal Plants, Phytochemicals and Therapeutics) IMPPAT (https://cb.imsc.res.in/imppat/home) database [36]. The canonical SMILES (Simplified Molecular Input Line Entry System) of each active compound were searched in the PubChem database. The drug likeness parameters were predicted using the Lipinski rule (molecular weight ≤500 g, hydrogen bond acceptor ≤10, hydrogen bond donor ≤5, calculated logP >5 or MlogP >4.15) and the Abbott bioavailability score [37]. The *in silico* ADME screening and drug-likeness evaluation was carried out using the web tool SwissADME (http://www.swissadme.ch/). The components that did not pass the screening were removed from the list. The filtered compounds were submitted to the Swiss Target Prediction server (http://www.swisstargetprediction.ch/) to identify the possible targets.

#### 2.1.2 Prediction of osteoarthritis-related target genes

The keyword “Osteoarthritis” was used to retrieve OA-related target genes. The Gene expression profile datasets GSE55457, GSE12021 and GSE55235 were downloaded from Gene Expression Omnibus database (https://www.ncbi.nlm.nih.gov/geo/). The GSE55457 dataset has 10 normal and 10 OA individuals, the GSE12021 dataset has 9 normal and 10 OA individuals, and the GSE55235 dataset has 10 normal and 10 OA individuals. The Differentially Expressed Genes (DEGs) of all three respective datasets were obtained for the normal and OA patients using iDEP 0.96 online tool. Genes meeting cut-off values p < 0.05 and |log FC| ≥ 0.5 were screened as DEGs between normal and OA individuals. The R package "limma" in iDEP.96 was employed for filtering up and down-regulated DEGs. The iDEP.96 online tool was used for all three datasets to visualise the volcano plots and heatmaps containing the 100 most significant genes each.

Four disease databases, namely GeneCards (https://www.genecards.org/), DisGeNET (https://www.disgenet.org/), OMIM (https://omim.org/), and TTD (https://db.idrblab.net/ttd/) were used to retrieve OA-related targets. After eliminating duplicate targets, all the OA-related target genes obtained from the three GEO datasets and disease databases were compiled into a single data file for further analysis.

#### 2.1.3 Constructing a network of compound-disease-target network

The Cytoscape v3.9.1 software is used to and visualise the relationship between each gene with active constituents and associated disease. The nodes indicate the herb, candidate active constituents, disease name and common targets, whereas the edges indicate the interconnection between the nodes. The degree of a node indicates the number of interactions it has with the nodes.

#### 2.1.4 Protein-protein interaction of common targets

The Venn diagram was constructed to obtain intersecting genes between targets of VTE and OA-associated genes, which might be potential targets for treating OA using the web tool Venny 2.0.2 (https://bioinfogp.cnb.csic.es/tools/venny/). Then, these common genes were uploaded to STRING v11.5 to construct a protein-protein interaction (PPI) network. The species option was set as “*Homo sapiens*”, and a high threshold score of 0.7 was selected. Disconnected nodes were removed from the network. The network was exported to Cytoscape v3.9.1 for visualisation. CytoHubba, a plugin of Cytoscape, was used to filter topologically important genes based on their ranking methods, i.e degree, maximal clique centrality (MCC) and maximum neighbourhood component (MNC) score. The Top 10 genes on the basis of score were selected as hub genes for subsequent studies.

#### 2.1.5 GO and KEGG pathway enrichment analysis

The intersecting common targets were used to analyse Gene Ontology (GO) annotation and Kyoto Encyclopedia of Genes and Genome (KEGG) analysis to understand the biological pathways associated with OA. KEGG enrichment analysis was performed using ShinyGo v0.77 (http://bioinformatics.sdstate.edu/go/) web server to identify the metabolic pathway associated with target genes. GO analysis was conducted using the DAVID database (https://david.ncifcrf.gov/) as it gives information about the biological pathways (BP), cellular components (CC) and molecular functions (MF). The visualisation of the enriched GO categories was done using an SR plot in the form of a bar graph. The significant pathways and functions were determined based on the threshold limit of p-value <0.05, FDR (False Discovery Rate) < 0.01 and by selecting the species as “Human”.

#### 2.1.6 Molecular docking

The molecular docking was performed between ten hub genes obtained from the PPI analysis and the top four active compounds from the compound-target-disease network. RCSB PDB database was used to download the crystal structure of each of the proteins in the PDB format. The PBD IDs for the proteins having no mutation and low resolution including BCL2 (PBD ID: 7LH7), FGF2 (PBD ID: 6L4O), ICAM 1 (PBD ID: 2OZ4), MAPK1 (PBD ID: 4ZZN), MMP1 (PBD ID: 3SHI), MMP2 (PBD ID: 7XJ0), MMP9 (PBD ID: 4WZV), COX2 (PBD ID: 5F1A), STAT3 (PBD ID: 6NJS) and ESR1 (PBD ID: 1 × 7R) were selected. The active site of the receptor protein with a higher pocket score was selected using the online server Prankweb (https://prankweb.cz). The receptor molecule was processed by removing water molecules, original ligand molecules and addition of non-polar hydrogen with the help of BIOVIA Discovery Studio Visualizer. The Swiss PDB view was used to fill the missing amino acid residues. The final preparation involves the addition of polar hydrogen, gasteiger charges, and energy minimization of each protein, which was done in UCSF Chimera. The two dimensional structure (2D) structure of the core compound was obtained from the PubChem database in SDF format. Then, the refined core proteins and core compounds were uploaded to the PyRx tool for docking. Autodock tool was used to minimise the energy of the ligand molecules and conversion them to pdbqt files. Finally, the docking visualisation was performed using Autodock Vina. The suitable X, Y and Z coordinates were set for each complex in the grid box as per the pocket-1 value given in the Prankweb for active site binding of the ligand. After docking, the binding affinity score was extracted to log*.txt files and the best-docked interactions were again visualized in Discover studio BIOVIA. The validation of docking was confirmed based on low RMSD (<2 Å) of the redocked ligand from the orientation of the co-crystallized ligand and the reproduction of observed interactions from the pdb structure. The best docking position was the one with the minimum root mean square deviation (RMSD) predicted by X-ray crystal configuration, and the binding energy between ligand and receptor protein was evaluated to indicate the binding strength.

#### 2.1.7 Molecular dynamics simulation

The molecular dynamic simulation was performed for top-docked complexes using the GROMACS package available in the SiBioLead LLC (https://sibiolead.com). The simulation pre-processing step was done to generate the topology of protein and ligand using AMBERTOOLS and ACPYPE tool kit. The complex was solvated in a triclinic-type periodic boundary box using a SPC water model. Before MD simulation, 0.15 mM of Na^+^/Cl^−^ ions were added to make the system electrically neutral. Next, the energy minimization process was done with a 10000 steps steepest descent algorithm. The simulation system was equilibrated using the constant volume**/**constant pressure method with an equilibration time of 100 ps. The equilibrium temperature was maintained at 300 K and pressure at 1 bar throughout the simulation. Finally, the MD simulation runs of the complexes was performed with a leap-frog integrator for 100 ns each, and the interpretation of the simulation result was made under GROMACS built-in tools.

### 2.2 In-vitro analysis

#### 2.2.1 Plant material and extract preparation

Fresh aerial roots of *Vanda tessellata* were collected from Machhaghara, Gajapati district in Odisha (19° 11′ 42.252″ N, 84° 19′ 42.276″ E, 544. 8 m above the sea level) and identified by taxonomist Prof. P.C. Panda. A voucher specimen (2536/CBT Dt. September 25, 2023) was deposited to the herbarium of SOA University. The roots were shade-dried for about 15 days until the moisture was removed and ground in an mechanical homogenizer. The extract was prepared from the dried powder (30 g) with the help of a soxhlet apparatus using methanol (200 ml) at temp. of 25 °C for 48 h. The extract was filtered using Whatmann filter paper and concentrated using a rotary vacuum evaporator (Hei-VAP Core HL, Heidolph, Schwabach, Germany). The extract yield was found to be 13.15 % on dry weight basis.

#### 2.2.2 Cell culture

The human synovial sarcoma cell line (SW-982) was purchased from NCCS, Pune, India. Cells were cultured in Dulbecco's Minimum Essential Medium (DMEM) with 10 % FBS, 1 % antibiotic-antimycotic solution and 1 % L-glutamine (200 mM). Cells were maintained in the CO_2_ incubator at 37^o^C in an atmospheric condition of 5 % CO_2_ and 18–20 % O_2_.

#### 2.2.3 Cell viability and migration analysis of SW982 cells

The MTT analysis was carried was to check the cytotoxic effect of the drug on the SW982 cell. The SW982 cells were seeded in the 96 well plates at 1 × 10^4^ cells/well and treated with VTE of various doses (6.25–200 μg/ml) for 24 h. The MTT solution was added to each well, followed by the replacement of DMSO (100 μl) after 3 h to solubilise the formazan crystals. The percentage of cell proliferation was checked by measuring the absorbance with a microplate reader at 570 nm.

Cells were seeded in 6 well plates at a density of 2 × 10^5^ cells/well until it attained 80 % confluence as a monolayer. After 24 h of incubation, the monolayer was gently scratched using a sterile pipette tip across the centre of the well. This was done without changing the media to create a wound. After scratching, the well was washed twice with medium to remove the detached cells. Then, the cells were treated with 10 ng/ml of IL-1β followed by different concentrations of VTE (50 and 100 μg/ml) and incubated at 37^o^C for 48 h. The cell images were captured at different intervals (0, 24 and 48 h). The microscope (Olympus CKX41-A32PH, Tokyo, Japan) was used for capturing the image of different views of the monolayer. The gap distance can be calculated using Image J software. The rate of wound closure was measured as follows:

Wound closure (%) = (Initial wound area at 0 h-Final wound area at t h)/Initial wound area at 0 h*100.

#### 2.2.4 Enzyme-linked immunosorbent assay

Quantitative ELISA was carried out to measure he expression levels of the pro-inflammatory cytokines. For this analysis, SW982 cells were cultured in 12-well plated and incubated for 48 h for cell attachment, followed by incubation for 2 h with IL-1β (10 ng/ml) to induce inflammation. Subsequently, the cells were treated with different doses of VTE (50 and 100 μg/ml) and incubated for 24 h. After the treatment, the supernatant was collected, and ELISA was performed to quantify the secretion level of PGE2, IL-6, IL-8 and TNF-α following the manufacturer's kit (RayBiotech, Norcross, GA).

#### 2.2.5 RT-qPCR analysis

The SW982 cells were plated in a 6-well plate at a density of 2 × 10^5^ cells/well and incubated at 37 °C for 24 h. Then, cells were stimulated with IL-1β (10 ng/ml) and incubated with different concentrations of VTE (6.25 & 25 μg/ml) for 24 h. The RNA was isolated from the cells using a Qiagen RNase kit as per the manufacturer's protocol. The complementary DNA (cDNA) was carried out using an IScript cDNA synthesis kit (Bio-Rad, CA). RT-qPCR reaction was performed on the Quant Studio3 system using SYBR Green Mastermix. The primer sequences used for the expression study are listed in Table 1. The amplification conditions for 40 cycles were as follows: initial denaturation: 95^o^C for 5 min, denaturation at 95^o^C for 10 s, annealing at 60 for 20 s and extension at 72^o^C for 20 s. The relative expression fold change was calculated relative to β-actin using △△Ct method.

**Table 1:** Oligonucleotide sequence of primers used for RT-qPCR analysis.

Gene names		primer Sequence
MMP-2	Forward	ACCTGGATGCCGTCGTGGAC
	Reverse	TGTGGCAGCACCAGGGCAGC
MMP-9	Forward	CAGTACCGAGAGAAAGCCTATT
	Reverse	CAGGATGTCATAGGTCACGTAG
β- actin	Forward	GGAGATTACTGCCCTGGCTCCTA
	Reverse	GACTCATCGTACTCCTGCTTGCTG

#### 2.2.6 Nuclear translocation assay of NF-κβ

SW982 cells (2.5 x 10^5^ cells/well) were cultured in a 96-well plate in CO_2_ for 24 h. Then, cells were stimulated with IL-1β (10 ng/ml) and kept for 2 h, followed by treatment of VTE (50 & 100 μg/ml). Further, 0.5 mL of BD Cytofix/Cytoperm solution was added, followed by washing with 0.5 % bovine serum albumin (BSA) in 1X phosphate-buffered saline (PBS) and 0.1 % sodium azide. Then, the cells were immunostained with 10 μL of PE Mouse anti–NF– κβ p65 antibody for 30 min and counter-stain with 100 μL of DAPI solution (1 μg/ml) for 10 min. The cells were imaged using the ZEISS LSM 880 imaging system (Carl Zeiss, Oberkochen, Germany) and NF-κβ p65 expression intensity was measured using software Image J.

#### 2.2.7 Detection of MAPK and STAT3 phosphorylation levels by ELISA

SW982 cells were grown to confluence in 24-well plates. The cells were stimulated with IL-1β (10 ng/ml) followed by treatment in the absence or presence of VTE (50 & 100 μg/ml) and incubated for 24 h. Subsequently, total and phosphorylated MAPKs (ERK 1/2, p38, and JNK) and STAT3 were detected from cell lysate using a cell-based ELISA kit (RayBiotech, Norcross, GA, USA) following the manufacturer's instructions.

## 3 Results and discussion

### 3.1 Network pharmacology

#### 3.1.1 Screening of drug-like candidates of Vanda tessellata and prediction of OA-related target genes

Thirteen active components of *Vanda tessellata* were identified from the literature [35] and the IMPPAT database. All these components successfully passed the ADME screening based on Lipinski's rule of five. This rule use to assess the drug-likeness of a compound, helps in predicting its absorption, distribution, metabolism, and excretion (ADME) properties. Larger molecules tend to have difficulty in crossing the membrane which decreases their bioavailability. High log P value indicates high lipophilicity with poor water solubility resulting in an inefficient proper absorption and distribution. Hydrogen donor or acceptor increases the water solubility but reduces its ability to pass lipid membrane thereby reducing the absorption of the compound [37] (Table 2). Compounds that comply with these rules are more likely to have good oral bioavailability, which is essential for oral medications. By filtering out compounds that are less likely to be orally active, compounds with a higher probability of success can be selected, thereby reducing attrition rates in drug development [38].

**Table 2:** Drug likeness screening of *Vanda tessellata* constituent.

Sl no.	Compounds	MW (g/mol)	ML OGP	HBA<10	HBD<5	Rotational bond<10	TPSA	WLOGP	Lipinski violation	Abbott Bioavailability
							(in Å^2^)			
1	β-Sitosterol	414.71	6.73	1	1	6	20.23	8.02	1	0.55
2	Daucosterol	576.85	3.96	6	4	9	99.38	5.85	1	0.55
3	Heptacosane	380.73	8.86	0	0	24	0	10.78	1	0.55
4	1-Octacosanol	410.76	7.07	1	1	26	20.23	10.14	1	0.55
5	Tessalatin	270.28	1.87	4	2	1	58.92	2.61	0	0.55
6	Gigantol	274.31	2.26	4	2	5	58.92	2.9	0	0.55
7	Gallic acid	170.12	−0.16	5	4	1	97.99	0.5	0	0.56
8	Gamma-Sitosterol	414.71	6.73	1	1	6	20.23	8.02	1	0.55
9	Stigmasterol	412.69	6.62	1	1	5	20.23	7.8	1	0.55
10	Dihydroconiferyl dihydro-p-coumarate	330.37	2.61	5	2	9	75.99	3.22	0	0.55
11	Methyl Linoleate	294.47	4.7	2	0	15	26.3	5.97	1	0.55
12	Syringaldehyde	182.17	0.24	4	1	3	55.76	1.22	0	0.55
13	Vanillin	152.15	0.51	3	1	2	46.53	1.21	0	0.55

A total of 1443 potential targets corresponding to these active compounds were identified with the canonical SMILES in the Swiss Target Prediction database (Table S1). After removing duplicates a total of 535 targets related to VTE was obtained.

**Fig. 1:**
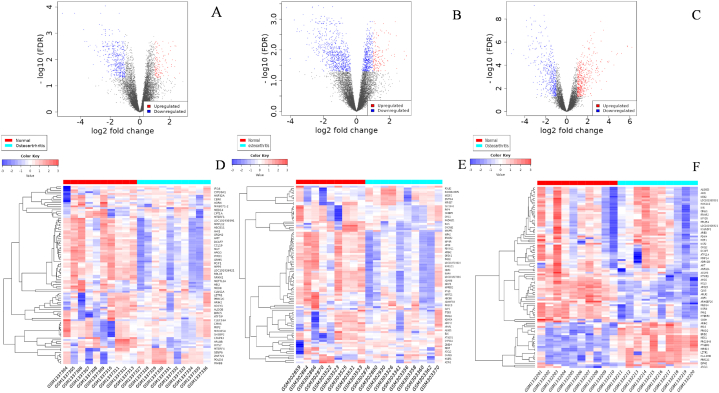
Gene expression levels between normal and Osteoarthritis patients were taken from the Gene Expression Omnibus (GEO) database. Heat map of top 100 up and down-regulated genes from the GEO Dataset (A) GSE55457, (B) GSE12021, and (C) GSE55235. The red and blue colour gradient indicates the up and down regulatory genes. The legend with the color key represents the log fold change of DEGs. Volcano plot distribution for GEO datasets (D) GSE55457 (E) GSE12021 (F) GSE55235. Red indicates highly expressive genes, whereas blue indicates less expressive genes of OA. (For interpretation of the references to colour in this figure legend, the reader is referred to the Web version of this article.)

The gene expression data of 29 individuals with normal joint conditions and 30 patients with osteoarthritis (OA) were obtained from the GEO dataset (GSE55457, GSE12021, and GSE55235). Differentially expressed genes (DEGs) between individuals with normal joint conditions and those with osteoarthritis (OA) were identified using the iDEP online tool. A log2 (Fold Change) threshold of 0.5 was used as a threshold value for analysing meaningful changes in gene expression. This threshold is suitable when looking for subtle alterations in expression. Several researchers have made use of log FC of 0.5 as a cut off criteria for selecting target genes with significantly differential expression [39,40]. Following a comparative analysis using volcano plots, 119 genes from GSE55457, 134 genes from GSE12021, and 458 genes from GSE55235 were identified to be upregulated in OA patients compared to normal individuals. Additionally, 433 genes from GSE55457, 486 genes from GSE12021, and 455 genes from GSE55235 were found to be downregulated. (Fig. 1A–C and Table S1). The heatmap displaying the top 100 DEGs of all three datasets are demonstrated where red represents normal patients and turquoise blue represents OA patients (Fig. 1D–F).

Furthermore, comprehensive searches were carried out in GeneCards, DisGeNET, TTD, and OMIM databases to collect genes associated with osteoarthritis (OA), resulting in the acquisition of 837 genes. After removing duplicate genes, a total of 2577 genes related to OA were filtered out. Venn diagram analysis resulted in 149 common intersecting genes between VTE targets and OA-related genes, suggesting promising key therapeutic targets for OA treatment.

#### 3.1.2 Constructing a network of drug-compound-disease-target interactions for the treatment of OA

**Fig. 2:**
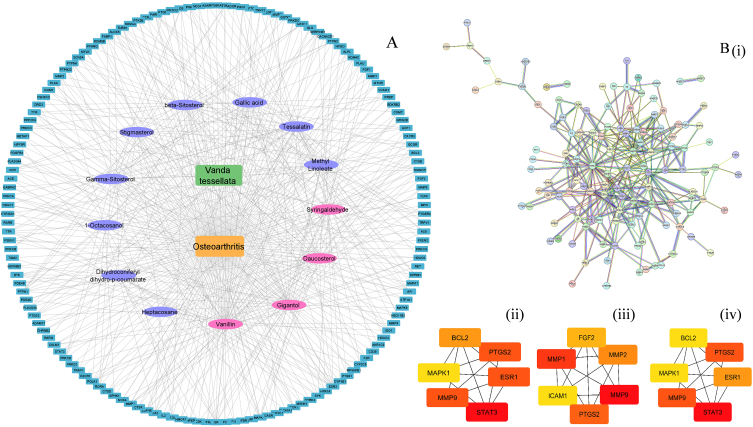
*Vanda tesselata* extract (VTE)and osteoarthritis (OA) targets. (A) The compound-target-disease network constructed by Cytoscape v_3.9.1 linking the OA-related targets with the compounds of VTE. The teal blue rectangular nodes indicate the target genes, Purple oval nodes indicate components of VTE with a lower degree score and the pink oval nodes components with the target genes, the purple oval nodes indicate components of VTE with a lower degree score, and the pink oval nodes are components with a high degree score.The (B) (i) Protein-protein interaction (PPI) network analysis between 149 common targets obtained from the STRING database. The of hub genes after topological screening on basis of (ii) degree (iii) MCC (iv) MNC plotted using Cytohubba. (For interpretation of the references to colour in this figure legend, the reader is referred to the Web version of this article.)

The relationship between VT compounds and their targets in Osteoarthritis treatment was visualized by constructing a compound-target-disease network using Cytoscape 3.9.1. The network comprises of 162 nodes and 487 edges. (Fig. 2A). The key active constituents among the identified compounds were filtered out on the basis of their degree score (>31). The top four compounds with degree value > 31 were vanillin, daucosterol, gigantol and syringaldehyde. They were selected as core compounds for the treatment against OA. Previous studies on the carrageenan-induced hind paw edema model have revealed that vanillin, syringaldehyde, and gigantol possess anti-inflammatory properties [30]. Daucosterol has therapeutic potential in the treatment of colitis due to its anti-inflammatory properties [41]. In addition, daucosterol demonstrates antioxidant, anti-apoptotic, neuroprotective, anti-diabetic, and immunomodulatory properties [[42], [43], [44]].

#### 3.1.3 Protein-protein interaction of common targets

The 149 intersecting targets were input into STRING database. After removing the disconnected nodes, generated network was exported to Cytoscape 3.9.1 software included 71 nodes and 348 edges as shown in Fig. 2B. The CytoHubba plug in was used for topological screening and selecting top 10 hub genes (BCL2, FGF2, ICAM 1, MAPK1, MMP1, MMP2, MMP9, COX2, STAT3 and ESR1) basing on different ranking criteria such as degree, MNC and MCC. An elevated level of BCL2 was observed in chondrocytes adjacent to osteoarthritic cartilage, which is significant as the protein promotes apoptosis in articular cartilage [45]. When apoptosis rate increases in chondrocytes, it will lead to cartilage tear and progression of OA [46]. FGF2 (Fibroblast growth factor-2) is a growth factor known for its catabolic effect in human articular cartilage, leading to its destruction [47,48]. Elevated levels of ICAM-1 expression in OA joints are associated with increased level of inflammatory mediators likes IL-6 and PGE2 [49]. The level of MMPs (MMP1, MMP2, and MMP9) are higher in individuals with OA as compared to those without, leading to matrix degradation [50,51]. COX-2 expression is increased in the synovial tissues of OA joints compared to healthy ones [52]. This upregulation is associated with increased production of prostaglandins, contributing to inflammation and pain in OA [53]. STAT3 is activated by inflammatory mediators such as IL-6, IL-1β and TNF-α in arthritic joints [54]. A study using the DMM mice model suggested that blocking STAT3 can reduce joint injury and pain in OA [55]. In OA, damaged cartilage is seen to produce a higher ESR1 compared to healthy cartilage. Knockdown of the ESR1 gene leads to upregulation of OA-relevant genes [56].

#### 3.1.4 Gene Ontology and KEGG analysis

**Fig. 3:**
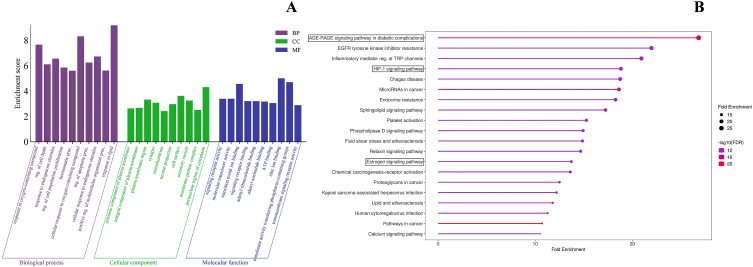
GO and KEGG pathway enrichment analysis of the intersecting targets of VTE and OA. (A) Bar plot of top 10 GO terms enrichment analysis of the biological process, cellular components and molecular functions, respectively. (B) Bubble plot showing the top twenty KEGG enrichment pathways of 149 common targets for OA.

Gene Ontology (GO) annotation and Kyoto Encyclopedia of Genes and Genome (KEGG) analysis were carried out on intersecting targets to understand the biological functions of the target genes in OA. GO enrichment analysis from the DAVID database showed that the candidate targets were involved in 689 GO terms, including 488 BP, 121 MF and 80 CC. The top 10 GO terms from BP, MF and CC were plotted (Fig. 3A). According to the BP result, the hub targets are mostly involved in the regulation of cell death, regulation of apoptotic processes and response to endogenous stimuli. Apoptosis, or programmed cell death, is known for modulating homeostasis in articular cartilage where excessive production was observed in injured cartilage [57]. In the cartilage of ageing populations, very high cell loss is observed due to apoptosis [58]. The CC terms involved intrinsic components of the plasma membrane, integral components of the plasma membrane, plasma membrane region, synapses, mitochondria, etc. The signalling receptor activity, molecular transducer activity, transition metal ion binding, signalling receptor binding, etc. are some MF that involves highest number of target genes.

A total of 145 terms was obtained using the DAVID database for KEGG pathway enrichment analysis, and the top 20 pathways on the basis of their –logP values were demonstrated in the bar graph (Fig. 3B). The analysis revealed that the principal pathway involved in the treatment of OA included the AGE-RAGE signalling pathway in diabetic complications, the HIF-1 signalling pathway, the Estrogen signalling pathway, etc. The AGE-RAGE signalling pathway plays a crucial role in osteoarthritis (OA) pathogenesis. In chondrocytes of elderly individuals, the accumulation of advanced glycation end-products (AGEs) and the receptor for AGEs (RAGE) disrupt proper signalling, leading to increased production of chemokines, cytokines, and MMPs. This cascade accelerates cartilage degradation. Targeting the AGE-RAGE signalling pathway may provide a potential avenue for OA treatment [59,60].

The HIF-1 (Hypoxia-Inducible Factor 1) signalling pathway is another relevant pathway associated with OA [61].Studies have shown increased levels of the transcription factor HIF-1α in OA cartilage samples, along with higher expression of its target genes [62,63]Activation of HIF-1α, triggered by the hypoxic joint microenvironment, regulates the expression of genes involved in inflammation and angiogenesis [64,65]. The accumulation of HIF-1α is responsible for increased matrix deposition in the growth plate by producing type II collagen and MMPs [66,67].

Estrogen receptor α (ER α) encoded by ESR1 gene plays a crucial role in maintaining articular cartilage homeostasis through ESR signalling pathway. Estrogen can protect articular cartilage from damage during OA development by promoting chondrocyte autophagy [68]. Previous findings indicates a connection between estrogen deficiency and the onset and advancement of osteoarthritis (OA), impacting different tissues within joints, such as articular cartilage. In vivo studies involving mice with ER α knocked out have demonstrated severe cartilage damage, increased osteophyte formation, and fibrosis in the joint capsule. These findings suggest that ER α may significantly influence the development of OA [69].

#### 3.1.5 Molecular docking and molecular dynamic simulation study

**Fig. 4:**
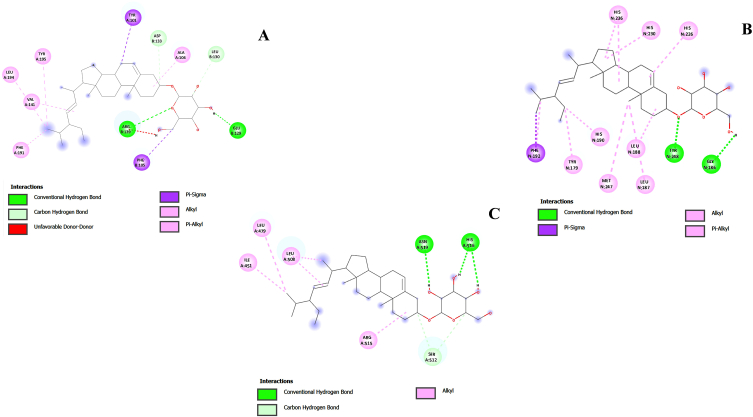
2D interaction of top docked complexes (A) BCL2_daucosterol complex (B) MMP9_daucosterol complex (C) ESR1_daucosterol.

Molecular docking was performed between ten hub genes obtained from the PPI analysis and the top four active compounds from the compound-target-disease network. The lower the binding energy, the higher the binding affinity, and the more stable the conformation. The BCL2-daucosterol complex showed the lowest binding energy of −10.2 kcal/mol, indicating the strongest interaction corresponds to the hydrogen bonds at the ASP 107, LEU 108, SER 106, and GLU 129 residues of BCL2. It also forms other interactions such as pi-sigma at THR 109 and PHE 105; alkyl and pi-alkyl at ALA 149, LEU 130, ALA 142, ALA 104, PHE 97, ARG 102, SER 145, GLU 98 residues (Fig. 4A). The binding energy of the BCL2-gigantol complex was −9.5 kcal/mol, with hydrogen bonds forming interactions with ASP 107, LEU 108, SER 106, GLU 129, and SER 145 amino acid residues. The current study aligns with previous docking studies that identified ASP 107 and LEU 108 as part of the active site of BCL-2 [70]. This study confirms that these residues are essential for the proper functioning of the protein, highlighting their critical role in binding energetics.MMP9showed a binding affinity of −10.1 kcal/mol with daucosterol, forming hydrogen bonds at GLY 186, TYR 248 and -9.3 kcal/mol with gigantol with hydrogen bonds at ALA 189, ALA 242, LEU 243, TYR 248 residues. MMP9 also interacted through pi-sigma, alkyl and pi-alkyl bonds with daucosterol at HIS 190, HIS 236, HIS 226, and PHE 192 residues (Fig. 4B).These interactions are similar to those reported by another researcher, where several compounds from the ZINC database were found to interact with multiple residues of the MMP-9 protein, including GLU 152, PHE 153, and VAL 133 [71]. Daucosterol exhibited hydrogen bonds with ASN 519, HIS 516, and SER 512 amino acid residues as well as alkyl bond at ILE 451, LEU 479, LEU 508, and ARG 515 residues of ESR1 (Fig. 4C), while gigantol formed hydrogen bonds at GLY 521, GLU 353, and LEU 387 residues, with binding energies of −10.1 and −9.3 kcal/mol, respectively. The obtained results corroborate the findings of a researcher, wherein isorhamnetin-ESR1 complex is seen to form hydrogen bond with LEU 387 amino acid residue [72]. The result of the dockings were visualise in the Discover studio, which indicated that the compounds and targets were mainly associated with hydrophobic interactions (Table 3).

**Table 3:** Binding affinities of receptor and ligand.

Protein	Ligand/inhibitor	Binding affinity (Kcal/mol)	No. of H-bonds	H-bond interaction	Other interactions
BCL2	Daucosterol	−10.2	4	ASP 107, LEU 108, SER 106, GLU 129	ALA 149,THR 109,LEU 130, ALA 142, ALA 104, PHE 105, PHE 97, ARG 102, SER 145, GLU 98
	Gigantol	−9.5	5	ASP 107, LEU 108, SER 106, GLU 129, SER 145	ALA 149, LEU 130, ALA 142, ALA 104, PHE 146, PHE 105, ARG 102, SER 145, GLU 98
	Syringaldehyde	−5.9	2	GLU 129, SER 106	LEU 130, ALA 142, ALA 104, PHE 105, PHE 97, PHE 146, LEU 108, ARG 102, PHE 105, PHE 97
	Vanilline	−5.8	2	SER 106, SER 145	ARG 102, PHE 146, ALA 142,LEU 130
MMP2	Daucosterol	−7.3	3	ASP 102, GLU 103, ASP 26	PRO 75, TRP 68, ASP 101, LYS 79
	Gigantol	−5.7	3	GLU 103, ASP 26, ASN 64	LEU 104, PRO 25, MET 62, ILE 21, HIS 98, ARG 67
	Syringaldehyde	−5.3		ILE 21, ILE, 20, ARG 53	TYR 23, PRO 29
	Vanilline	−5.3	2	ARG 53, ILE 54	TYR 23, ILE 20, TYR 55, PRO 29, ARG 19
MAPK1	Daucosterol	−8.6	5	MET 106, GLU 107, SER 39, SER 27, TYR 28	VAL 37, LEU 129, ILE 29
	Gigantol	−7.3	2	LYS 52, MET 106	VAL 37, ALA 50, LEU 154, ILE 29, GLU 107, CYS 164
	Syringaldehyde	−5	1	GLN 103	ILE 82, LEU 154, MET 106, ILE 29, ALA 50, VAL 37
	Vanilline	−5.6	2	TYR 62, ARG 65	THR 66
STAT3	Daucosterol	−6.8	1	THR 526	ALA505, TRP501
	Gigantol	−6.3	3	SER540, ASN538, GLU506	THR 526,TRP501, ALA505
	Syringaldehyde	−4.8	2	LYS548, LYS548	ALA547, LEU532, MET554, ALA555, GLU552
	Vanilline	−4.6	4	ALA555,GLU552, ASN553, ALA547	LEU532,MET552, LYS548
COX2	Daucosterol	−7.6	3	HIS 207, PHE 210, THR 212	VAL 291
	Gigantol	−2.5	–	TRP 139	
	Syringaldehyde	−5.9	3	TYR 385, SER 530, VAL 523	TRP 387, LEU 384, LEU 352, ALA 527, VAL 349
	Vanilline	−6	2	HIS 207, THR 206	GLN 203, TRP 387, TYR 385
ESR1	Daucosterol	−10.1	3	ASN 519, HIS 516, SER 512	ILE 451, LEU 479, LEU 508, ARG 515
	Gigantol	−9.3	3	GLY 521, GLU 353, LEU 387	LEU 525, HIS 524, PHE 404, LEU 391, LEU 387, GLU 353, ALA 350,
	Syringaldehyde	−5.9	4	MET 357, GLY 390, ARG 394, GLU323	HIS 356, LEU 327, PRO 324, PHE 445
	Vanilline	−6.1	3	ARG 214, ALA 182,HIS 218	HIS 228, VAL 215, LEU 181, GLU 219, TYR 237, TYR 240
FGF2	Daucosterol	−6.3	2	VAL 204, GLY 203	LEU 282, PRO 283
	Gigantol	−5.7	1	ARG 249	ARG 239, GLU 241
	Syringaldehyde	−4.5	1	ARG 249	ILE 279, TYR 257, PRO274, THR 247, ARG 239, PHE 237
	Vanilline	−4.2	3	ARG 249, THR 24, GLU 241	ARG 239, TYR 257
MMP 9	Daucosterol	−10.1	2	GLY 186, TYR 248	HIS 190, HIS 236, HIS 226, PHE 192
	Gigantol	−9.3	4	ALA 189, ALA 242, LEU 243, TYR 248	HIS226, VAL 223, LEU 222
	Syringaldehyde	−5.9	5	TYR 245, ALA 189, TYR 248, LEU 22, GLU 227	HIS 236, HIS 226, VAL 223, LEU 188
	Vanilline	−6.1	0	–	TYR 248, LEU 222, VAL 223, HIS 226, MET 247

The molecular docking results were validated through molecular dynamics (MD) simulation analysis. The MD simulation was performed for high ranked docked complex, i.e. for three selected apoproteins (BCL2, ESR1 and MMP9) and their interaction with two lead compounds, daucosterol and gigantol, over a duration of 100 ns. Several parameters such as root mean square deviation (RMSD), root mean square fluctuation (RMSF) analysis, solvent accessible surface area (SASA), radius of gyration (Rg), and change in secondary structure were assessed during the MD simulations to evaluate the configurational stability of both proteins and ligands. Furthermore, binding free energy calculations using the MM/PBSA (Molecular Mechanics/Poisson-Boltzmann Surface Area) method were performed to quantify the thermodynamic stability of the docked complexes.

**Fig. 5:**
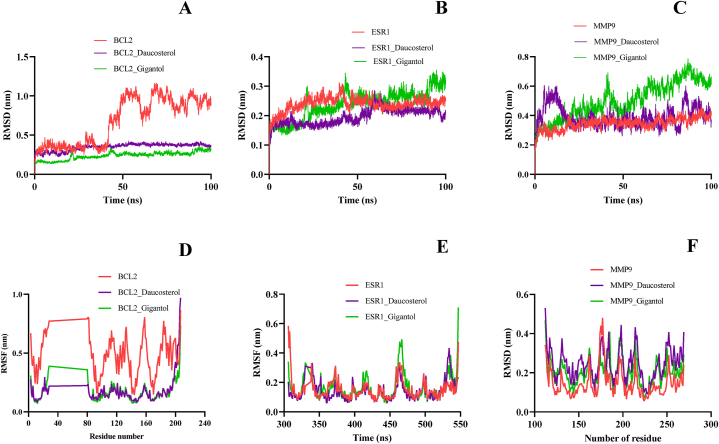
RMSD plot obtained during MD simulation of (A) apo-BCL2, BCL2-daucosterol and BCL2-gigantol (B) apo-ESR1, ESR1-daucosterol and ESR1-gigantol (C) apo-MMP9, MMP9-daucosterol and MMP9-gigantol. RMSF plot obtained during MD simulation of (C) apo-BCL2, BCL2-daucosterol and BCL2-gigantol (D) apo-ESR1, ESR1-daucosterol and ESR1-gigantol (F) apo-MMP9, MMP9-daucosterol and MMP9-gigantol complex.

The stability of the receptor-ligand complex was analysed by measuring the root mean square deviation (RMSD) of backbone atoms. The fluctuation in the protein can be interpreted by RMSD curve [73]. The plot shows that both the BCL2-daucosterol and BCL2-gigantol complexes were significantly stable throughout the simulation, indicating that both complexes remained firmly attached to their corresponding protein molecules without dissociating from the pocket during the simulation (Fig. 5A).The mean RMSD value of the ESR1-daucosterol complex was 0.194 nm, while for the ESR1-gigantol complex, it was 0.237 nm. This suggests that ESR1-daucosterol had greater stability during the course of the simulation (Fig. 5B). Initially, the MMP9-daucosterol complex showed fluctuation around 0.3 nm in between 5 and 20 ns, then became stabilized to the end of the simulation with some negligible jumps at around 80 ns. MMP9-gigantol complex was initially stable up to 40ns. After 40 ns, the fluctuation of the RMSD plot indicates that there must have been protein folding that has resulted in conformational and structural changes within the complex (Fig. 5C).

The RMSF plot indicates that the docked complex of BCL2 is more stable than undocked protein (Fig. 5D). Notably, there is a consistent region between residues 28–80 where the plot remains constant, indicating a missing or disordered structure contributing to instability. In the case of ESR1, ESR1-daucosterol and ESR1-gigantol, a similar fluctuation pattern was observed (Fig. 5E). However, ESR1-gigantol demonstrates higher flexibility at two specific regions: residues 412–420 and 455–474. In the case of the MMP9-daucosterol complex, there is greater flexibility observed at residues 129–162 and 249–269 compared to the MMP9-gigantol complex (Fig. 5F)

**Fig. 6:**
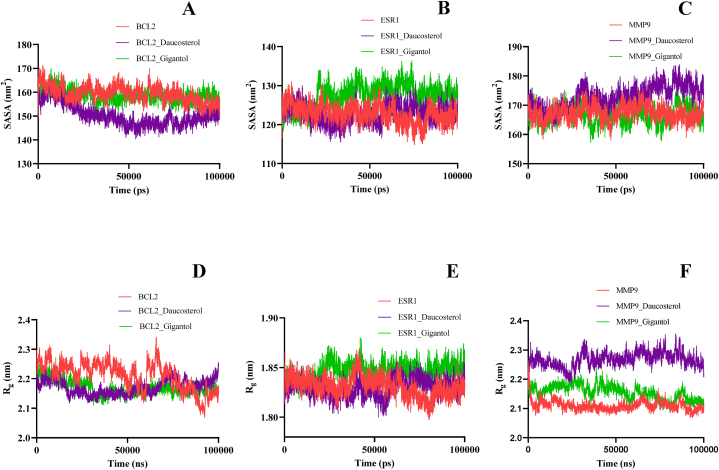
SASA plot of (A) apo-BCL2, BCL2-daucosterol and BCL2-gigantol (B) apo-ESR1, ESR1-daucosterol and ESR1-gigantol (C) apo-MMP9, MMP9-daucosterol and MMP9-gigantol complex. Radius of gyration plot of (C) apo-BCl2, BCL2-daucosterol and BCL2-gigantol (D) apo-ESR1, ESR1-daucosterol and ESR1-gigantol (F) apo-MMP9, MMP9-daucosterol and MMP9-gigantol complex.

SASA analysis was performed to detect the degree of exposure of the receptor to surrounding solvent molecules during the simulation [74]. The average SASA values of BCL2-daucosterol (150.31 ± 3.65 nm^2^), ESR1-daucosterol (1.82 ± 0.01 nm^2^) and MMP9-gigantol (166.8183 ± 2.35 nm^2^) were lower than average SASA of their corresponding apo-protein BCL2 (159.39 ± 2. 74 nm^2^), ESR1 (183 ± 0.01 nm^2^) and MMP9 (167.34 ± 2.11 nm^2^). Similarly, the average SASA values of BCL2-gigantol, ESR1-gigantol and MMP9-daucosterol complexes was slightly higher than their respective apo-proteins. The trajectory of the protein and its complexes reveals that the solvent-accessible surface area gradually decreases in the course of the simulation, indicating that the bindings between them gradually increase (Fig. 6A–C).

The radius of gyration (Rg) is used to evaluate the structural compactness of a receptor-ligand complex. A lower extent of fluctuation suggests a higher degree of compactness which tends to higher stability [75]. As shown in Fig. 6D, BCL2-daucosterol and BCL2-gigantol had an average Rg of 2.17 nm and 2.16 nm, respectively. The comparative result suggests that BCL-2 exhibited more stable behaviour after binding with the complexes, thus indicating the structural compactness of the complexes. In the 100 ns of simulation trajectory, ESR1, ESR1-daucosterol and ESR1-gigantol showed a comparable trajectory with a stabilized Rg in the range of 1.82–1.84 nm (Fig. 6E). The mean Rg value of MMP9-gigantol was 2.16 nm, which is lower compared to MMP9-daucosterol (2.27 nm), hence concluding that gigantol formed a more stable and compact complex with MMP9 than that daucosterol (Fig. 6F).

**Fig. 7:**
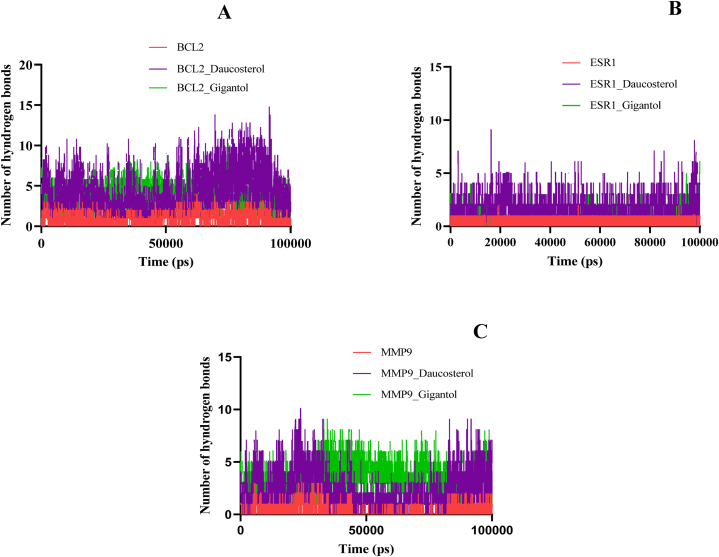
Intramolecular hydrogen bond analysis (A) apo-BCL2, BCL2-daucosterol and BCL2-gigantol (B) apo-ESR1, ESR1-daucosterol and ESR1-gigantol (C) apo-MMP9, MMP9-daucosterol and MMP9-gigantol complex.

The hydrogen bond is a strong, non-covalent interaction. The docked complex BCL2-daucosterol formed a maximum number of intramolecular hydrogen bonds in the range of 0–16. Similarly, the number of hydrogen bonds formed for BCL2-gigantol and MMP9-daucosterol complexes was 0–11 whereas for ESR1-daucosterol and MMP9-gigantol complex it was 0–10. The ESR1-gigantol docked complex showed 0–7 hydrogen bond, which is the lowest among all the complexes. The calculation of hydrogen bonds suggested that all six complexes were highly stabilized in the course 0–100 nm simulation (Fig. 7A–C).

**Fig. 8:**
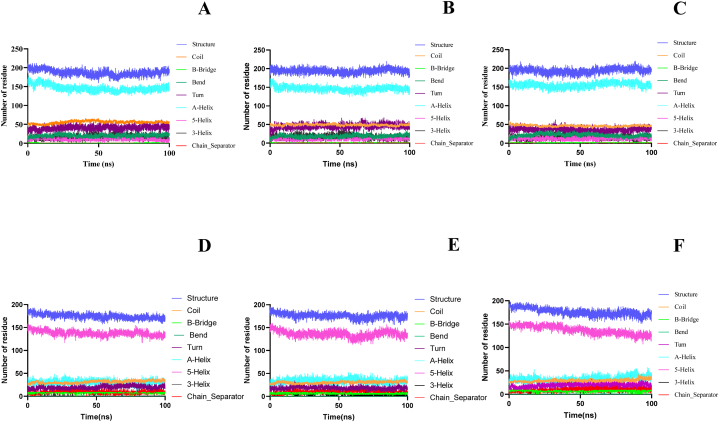
Secondary structure analysis of (A) apo-BCL2 (B) BCL2-daucosterol (C) BCL2-gigantol complex (D) apo-ESR1 (E) ESR1-daucosterol (F) ESR1-gigantol.

**Fig. 9:**
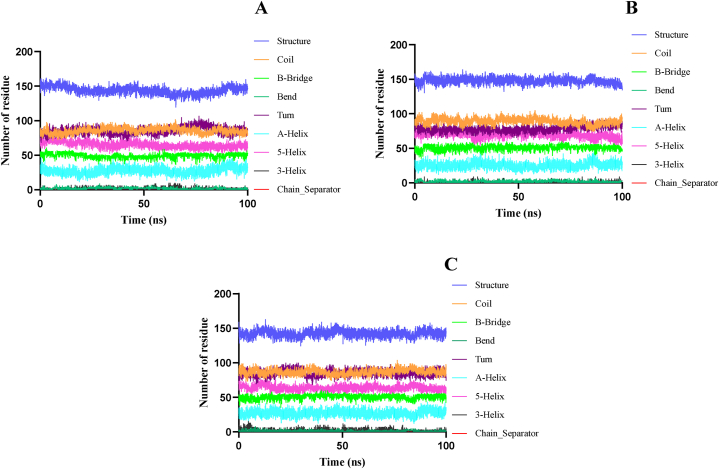
Secondary structure analysis of (A) apo-MMP9 (B) MMP9-daucosterol (C) MMP9-gigantol complex.

The secondary structure content was analysed to understand of the conformational changes in the secondary structure of the protein-ligand complex during the 100 ns simulation. As shown in Fig. 8A–F, no significant changes were observed in the secondary structure of apo BCL2 and ESR1 protein and its complexes. MMP9 showed fluctuation in coils and turns after binding with both daucosterol and gigantol (Fig. 9A–C).

The MM/PBSA tool in GROMACS was used to calculate energy parameters of docked complexes (Table 4). The average binding affinity of docked complexes including BCL2_daucosterol BCL2_gigantol, ESR1_daucosterol, ESR1_gigantol, MMP9_daucosterol, MMP9_gigantol were −48.28, −32.83, −38.08, −36.22, −42.97 and −32.05 kcal/mol. Out of all the complexes, BCL2_daucosterol shown the most favourable average binding affinity of −48.28 kcal/mol. Conversely, MMP9_gigantol exhibited the least favourable average binding i.e, −32.05 kcal/mol.

**Table 4:** MMPBSA analysis of docked complex.

Complex	ΔVDWAALS	ΔEEL	ΔEPB	ΔENPOLAR	ΔEDISPER	ΔGGAS	ΔGSOLV	ΔTOTAL
BCL2_daucosterol	−65.2	6.6	28.66	5.13	0	−71.8	23.53	−48.28
BCL2_gigantol	−46.59	−4.75	21.86	−3.34	0	−51.34	18.52	−32.83
ESR1_daucosterol	−49.34	−1.77	17.04	−4.01	0	−51.11	13.03	−38.08
ESR1_gigantol	−45.68	−2.47	15.31	−3.38	0	−48.15	11.93	−36.22
MMP9_daucosterol	−62.49	−4.09	29.15	−5.54	0	−66.58	23.61	−42.97
MMP9_gigantol	−46.18	−8.24	25.7	−3.33	0	−54.42	22.37	−32.05

Notes: ΔVDWAALS, van der Waals energy; ΔEEL, Electrostatic energies; ΔEPB, Polar solvation energy; ΔENPOLAR, Nonpolar solvation energy; ΔGGAS (Gas Phase Gibbs Free Energy) = ΔVDWAALS + ΔEEL; ΔGSOLV (Solvation Gibbs Free Energy) = ΔEPB + ΔENPOLAR; ΔTOTAL (Total Gibbs Free Energy Change) = ΔGSOLV + ΔGGAS.

### 3.2 *In vitro* validation in SW982 cells

#### 3.2.1 Effect of VTE on viability and migration of SW982 cells

**Fig. 10:**
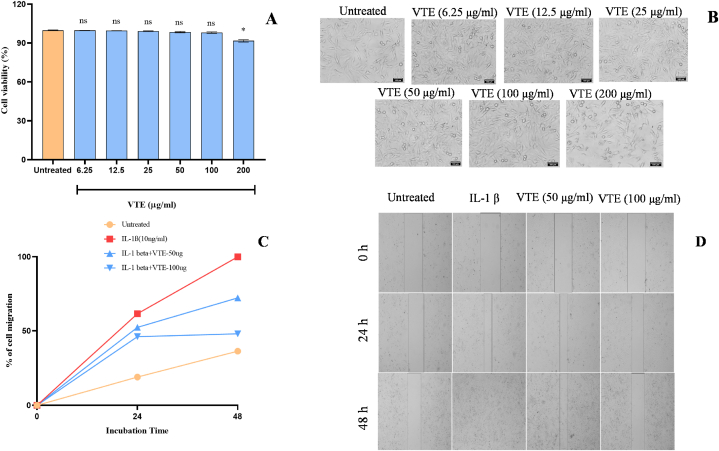
Effect of VTE on viability and migration of SW982 cells (A) Percentage of cell viability of VTE on SW982 cells. The orange bar represents the untreated cells, and the blue bar represents SW982 cells treated with different doses of VTE (6.25–200 μg/ml). The graph is plotted by integrating mean ± SD (n = 3). Statistical significance was calculated by using one-way ANOVA followed by the Tukey test. ^#^*p* < 0.05 between untreated and IL1β-induced group; ^ns^*p*>0.05, *p < 0.05 between IL-1β and VTE treated group. (B) The morphology was observed under a microscope and imaged (scale bar 50 μm). (C) Comparative scatter graph represents the wound healing effect of VTE against the IL-1β induced SW-982 cells at different intervals in terms of % wound closed area (D) wound healing activity of VTE at different time intervals of 0, 24 and 48 h on IL-1β induced SW982 cells along with controls. (For interpretation of the references to colour in this figure legend, the reader is referred to the Web version of this article.)

The cytotoxic activity of VTE against SW982 was measured by the MTT assay. The result suggests that VTE was non-toxic against the SW982 cells up to a concentration of 100 μg/ml after 24 h of treatment, while 200 μg/ml caused moderate toxicity. Therefore, 100 μg/ml of VTE was chosen as the highest concentration for subsequent assay, as at this concentration, the cell viability did not show significant effects compared to the untreated group. The cell morphology in untreated cells was spherical and regular. The treatment of cells with VTE up to 100 μg/ml did not result in cell spreading and pseudopodia development, indicating that the majority of the cells were alive (Fig. 10A and B). A previous study on mice models has also shown VTE to exhibit very low toxicity levels [29].

The effect of VTE on the migration of human synovial SW982 cells was examined by the scratch wound assay. The distances between the migration fronts of untreated and VTE-treated SW982 cells were significantly different at 0, 24 and 48 h. IL-1β stimulated cells exhibited significant migration at 24 h, followed by nearly complete wound closure at 48 h. In contrast, VTE effectively inhibited the migration rate in SW-982 cells in a time-dependent manner, with wound healing rates of 72.26 % and 48.04 % after 48 h of incubation at 50 and 100 μg/ml, respectively (Fig. 10C and D). These results confirmed that VTE effectively inhibited the proliferation and migration of IL-1β induced cells after 24 and 48 h of incubation.

#### 3.2.2 Effect of VTE on inflammatory mediators in SW982 cells

**Fig. 11:**
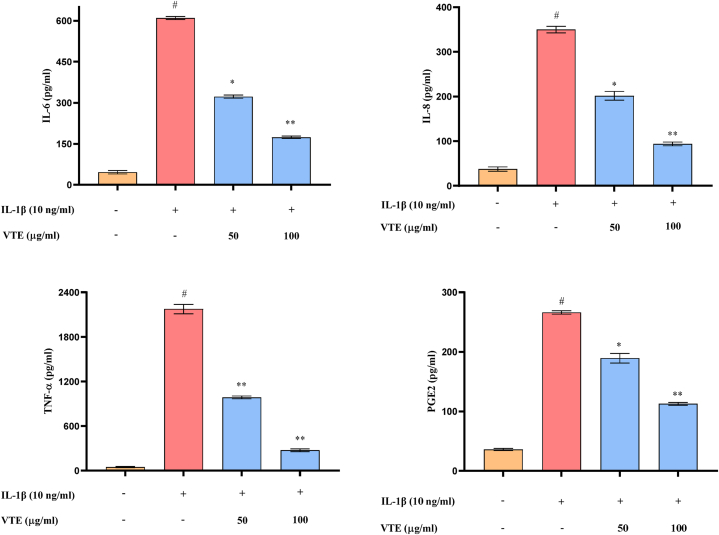
Effect of VTE on inflammatory mediators (A) IL-6, (B) IL-8, (C) TNF-α, (D) PGE2 in IL-1β induced SW982 cells by using ELISA assay. Cells treated with IL-1β (10 ng/ml) for 2 h followed by different doses (50 and 100 μg/ml) of VTE and incubated for 24 h. The graph is plotted by integrating mean ± SD (n = 3). Statistical significance was calculated by using one-way ANOVA followed by the Tukey test. ^#^*p* < 0.05 between untreated and IL1β-induced group; *p < 0.05 and **p < 0.01 between IL-1β and VTE treated group.

The pro-inflammatory mediators play a crucial role in the pathogenesis of OA [76,77] Several studies demonstrated an elevated amount of cytokines like IL-6, IL-8 and TNF- α in OA joints, leading to inflammation and cartilage degradation [78,79]. The elevated level of IL-1β stimulates the production of pro-inflammatory cytokines in articular cartilage indicating the correlation between inflammation and OA [80]. From this study, it was observed that the induction of IL-1β in the SW982 cells increased IL-6, IL8, PGE2 and TNF-α levels significantly (p < 0.05) to 92.29 % (13.07 fold), 89.27 % (9.32 fold), 86.43 % (7.36 fold) and 97.75 % (44.48 fold) respectively, as compared with the untreated group. A considerable reduction of 47.15 % (1.89 fold), 42.36 % (1.73 fold), 28.68 % (1.40 fold) and 54.68 % (2.20 fold) in the expression level of IL-6, IL8, PGE2 and TNF-α of SW982 cells were recorded when treated with VTE at 50 μg/ml. However, treatment with VTE at 100 μg/ml inhibited IL-6, IL8, PGE2 and TNF-α significantly by 71.63 % (3.51 fold), 73.17 % (3.72 fold), 57.66 % (2.36 fold) and 87.39 % (7.93 fold) respectively, compared to IL-1β treated group (Fig. 11A–D). These results confirm that VTE can downregulate the expression of multiple inflammatory mediators. Gigantol was reported to be an effective compound for suppressing IL-1β mediated inflammation in mice chondrocytes [81]. Therefore, we expect VTE to be an effective drug for treating against OA.

#### 3.2.3 Effect of VTE on the gene expression level in SW982 cells

**Fig. 12:**
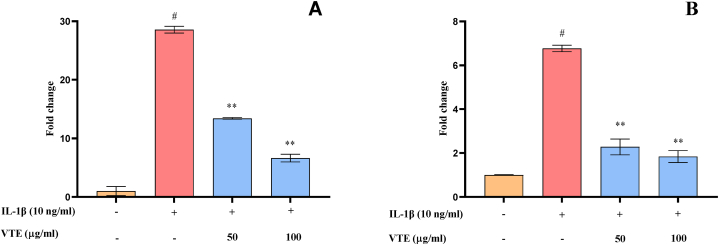
Effect of VTE on the expression level of MMPs. (A) MMP2 (B) MMP9 in IL-1β induced SW-982 cells. The graph has been generated by using the mean ± SD (n = 3). Statistical significance was calculated by using one-way ANOVA followed by the Tukey test. ^#^*p* < 0.05 between untreated and IL1β-induced group,*p < 0.05 and **p < 0.01 between IL1β and VTE treated group.

Matrix metalloproteinases (MMPs) play a crucial role in the destruction of articular cartilage [[82], [83]]. IL-1β induces chondrocytes to generate MMPs that inhibit the synthesis of the cartilaginous extracellular matrix in chondrocytes, resulting in the degradation of articular cartilages [83,84]. Additionally, chondrocytes express a variety of NF-κβ-mediated catabolic cytokines and chemokines that increase the production of MMPs while decreasing collagen and proteoglycan synthesis [85]. The mRNA expression level of matrix metalloproteinase genes (MMP2 and MMP9) was measured in SW982 cells. There was a significant reduction in the mRNA expression level of MMP2 by 2.27 and 1.84 fold in the VTE (50 and 100 μg/ml) treated group compared to the IL-1β induced group. Similarly, pre-treatment with VTE at doses of 50 and 100 μg/ml resulted in significant inhibition by 13.42 and 6.65 fold of MMP9 as compared to IL-1β stimulated group (Fig. 12A and B). Hence, it could be suggested that VTE may have the potential of to alleviate OA by reducing the level of MMPs.

#### 3.2.4 Effect of ENE on the nuclear translocation of NF- kβ in SW982 cells

**Fig. 13:**
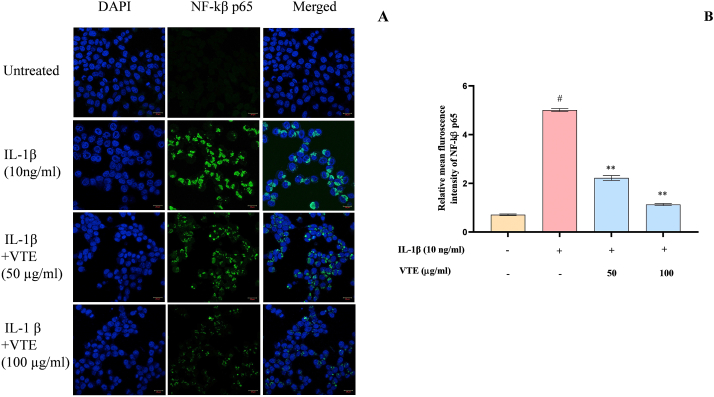
Effect of VTE on NF-kβ nuclear translocation in IL-1β induced SW982 cells. (A) Confocal microscopy image of NF-kβ. Cells stained with DAPI (blue), immunolabelled for NF-kβ (green) (B) The graph has been generated using mean ± SD (n = 3). Statistical significance were calculated by using one way ANOVA followed by Tukey test. ^#^*p* < 0.05 between untreated and IL1β-induced group; ^#^*p* < 0.05 between untreated and IL1β-induced group and **p < 0.01 between IL1β and ENE treated group. (For interpretation of the references to colour in this figure legend, the reader is referred to the Web version of this article.)

NF-κβ dimers are initially located in an inactive form in the cytoplasm, bound to I κβ subunit. Stimulated by a variety of chemical and mechanical signals leads to phosphorylation of I κβs, triggering the activation of NF- κβ [86]. Subsequently, the activated NF- κβ heterodimer translocate into the nucleus where it initiate the transcription of genes releasing chemokines, cytokines, angiogenic factors, proteases, etc [87]. These cytokines play a crucial role in the pathogenesis by inducing synovial membrane inflammation and articular cartilage degradation [83]. To verify the effect of VT on NF- κβ signalling, we measured the nuclear translocation of NF- κβ in SW982 cells (Fig. 13A). After treating with VTE at a concentration of 50 and 100 μg/ml, a decrease in fluorescence intensity of NF- κβ by 55.73 % (2.26 fold) and 77.4 % (4.43 fold), respectively, was observed as compared to IL-1β treated group (Fig. 13B). These results suggest that VTE effectively inhibited IL-1β induced NF- κβ signalling activation in SW982 human synovial cells. In animal models, injury-induced cartilage lesions were alleviated by the knockdown of NF- κβ p65 in the knee joints [88]. Gigantol has been reported of inhibiting NF- κβ pathway activation to protect mouse OA chondrocytes [81]. Hence, VTE can be effective in blocking the nuclear translocation of p65 subunit of NF- κβ and thus may have the potential to treat OA.

#### 3.2.5 Effect of VT extract on the MAPKs and STAT3 in SW982 cells

**Fig. 14:**
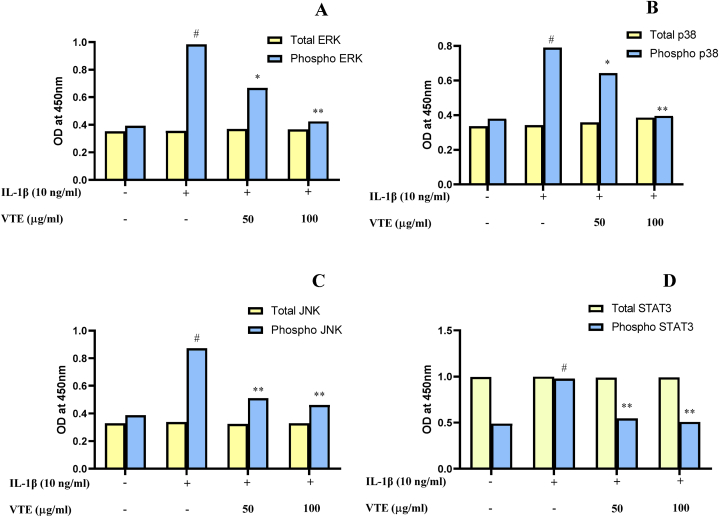
Effect of VTE on changes in phosphorylation of (A) ERK2 (B) p38 (C) JNK and (D) STAT3 in IL-1β stimulated SW982 cell by ELISA test. The graph has been plotted by using mean ± SD (n = 3). Statistical significance were calculated by using one way ANOVA followed by Tukey test. ^#^*p* < 0.05 between untreated and IL1β-induced group; *p < 0.05 and **p < 0.01 between IL1β and ENE treated group.

Activation of MAPKs (ERK, JNK, and p38) regulates the gene encoding chemokines, cytokines and proteases that contribute to the destruction of joint tissues especially through MMPs [13]. The signal transducer and activator of transcription 3 (STAT3) are triggered by multiple cytokines in the OA microenvironment [89]. Inhibition or knockdown of STAT3 can suppress articular lesions by reducing the proliferation, migration and angiogenesis of endothelial cells. *In vivo* studies on DMM mice suggest that the inhibition of STAT3 can reverse angiogenesis and subchondral bone damage [89]. Cell-based ELISA was performed to investigate whether the effect of VTE is attributed to the inhibition of the MAPKs and STAT3 activation. It was observed that the level of phosphorylation of MAPKs (ERK1, p38, and JNK) and STAT3 was elevated by 60.07 %, 52 %, 49.05 % and 49.90 % compared to the untreated group after induction of SW982 cells with IL-1β, thereby demonstrating the activation of kinases. However, after pre-incubation of IL-1β SW982 cells with VTE (50 and 100 μg/ml), the rate of phosphorylated ERK1, p38, JNK and STAT3 decreased by 32.10 % & 56.10 %, 18.66 % & 49.97 %, 41.46 % & 47.19 % and 44.10 %& 48 %respectively (Fig. 14A–D). Our findings demonstrated that VTE administration inhibited the MAP kinase pathway.

## 4 Conclusion

The current findings, for the first time, delve into the pharmacological and molecular mechanism of action of *Vanda tessellata* extract in treating osteoarthritis using system biology approaches. The multi-component and multi-pathway features of the phytoconstituents present in VTE, and their mechanism of action were elucidated. Despite significant advancements, the current research primarily relied on network pharmacology and *in vitro* experimental assays. However, further validation in *in vivo* animal models is necessary to confirm the safety and efficiency of the drug. The findings reveal that *Vanda tessellata* extract exerts strong activity against osteoarthritis by suppressing the expression level of inflammatory mediators, matrix metalloproteinases and inhibiting the activation of NF-κβ and MAPK pathways in SW-982 cells. This suggests it's potential as a promising candidate for developing novel drugs aimed at treating osteoarthritis.

## Funding

This study received no funds or any extramural research grants

## Data availability statement

Data will be made available on request.

## CRediT authorship contribution statement

**Sucheesmita Padhee:** Writing – original draft, Investigation, Data curation. **Debajani Mohanty:** Data curation. **Ambika Sahoo:** Data curation. **Sudipta Jena:** Data curation. **Pratap Chandra Panda:** Supervision. **Asit Ray:** Writing – review & editing, Methodology, Conceptualization. **Sanghamitra Nayak:** Writing – review & editing, Supervision, Funding acquisition.

## Declaration of competing interest

The authors declare that they have no known competing financial interests or personal relationships that could have appeared to influence the work reported in this paper.
